# Adamts10 controls transforming growth factor *β* family signaling that contributes to retinal ganglion cell development

**DOI:** 10.3389/fmolb.2022.989851

**Published:** 2022-09-06

**Authors:** Lauren K. Wareham, Amy E. Whitener, Hang-Jing Wu, Shu-Yu Wu, Hassane S. Mchaourab, Douglas P. Mortlock, Rachel W. Kuchtey, John Kuchtey

**Affiliations:** ^1^ Vanderbilt Eye Institute, Vanderbilt University Medical Center, Nashville, TN, United States; ^2^ Department of Molecular Physiology and Biophysics, Vanderbilt University, Nashville, TN, United States

**Keywords:** ADAMTS10, transforming growth factor beta (TGFβ), retinal development, zebrafish, morpholino (MO), retina, retinal ganglion cell (RGC)

## Abstract

Although mutations in *ADAMTS10* have long been known to cause autosomal recessive Weill-Marchesani Syndrome which is characterized by short stature and ocular abnormalities, more recent work has shown that certain mutations in *ADAMTS10* cause glaucoma in dogs. In humans, glaucoma is the leading cause of irreversible vision loss that affects tens of millions of people world-wide. Vision loss in glaucoma is a result of neurodegeneration of retinal ganglion cells that form the inner-most layer of the retina and whose axons form the optic nerve which relays visual information to the brain. ADAMTS10 contributes to the formation of microfibrils which sequester latent transforming growth factor *β* (TGFβ). Among its many biological functions, TGFβ promotes the development of retinal ganglion cells and is also known to play other roles in glaucoma pathogenesis. The aim of this study was to test the hypothesis that ADAMTS10 plays a role in retinal ganglion cell development through regulation of TGFβ signaling. To this end, Adamts10 expression was targeted for reduction in zebrafish embryos carrying either a fluorescent reporter that labels retinal ganglion cells, or a fluorescent reporter of pSmad3-mediated TGFβ family signaling. Loss of adamts10 function in zebrafish embryos reduced retinal ganglion cell reporter fluorescence and prevented formation of an ordered retinal ganglion cell layer. Targeting adamts10 expression also drastically reduced constitutive TGFβ signaling in the eye. Direct inhibition of the TGFβ receptor reduced retinal ganglion cell reporter fluorescence similar to the effect of targeting adamts10 expression. These findings unveil a previously unknown role for Adamts10 in retinal ganglion cell development and suggest that the developmental role of Adamts10 is mediated by active TGFβ family signaling. In addition, our results show for the first time that Adamts10 is necessary for pSmad3-mediated constitutive TGFβ family signaling.

## Introduction

Members of the ADAMTS family of secreted metalloproteases are highly conserved across vertebrate species and contribute to a variety of biological processes, including remodeling of extracellular matrix and development (Apte, 2009; Brunet et al., 2015; Kelwick et al., 2015). Previously, we identified a G661R mutation in *ADAMTS10* as disease-causative for a colony of Beagle dogs with autosomal recessive inheritance of glaucoma (Kuchtey et al., 2011). Protein structure modeling of the G661R mutation predicts disruption of normal ADAMTS10 folding (Kuchtey et al., 2011), though this does not prevent secretion of ADAMTS10 (unpublished data). Involvement of ADAMTS10 with glaucoma has been verified in another dog breed and extended to include ADAMTS17 which is structurally and functionally highly similar to ADAMTS10 (Kuchtey et al., 2013; Ahonen et al., 2014; Forman et al., 2015; Oliver et al., 2015; Karoulias et al., 2020b). In humans, glaucoma is the leading cause of irreversible vision loss affecting tens of millions of people world-wide. Vision loss in glaucoma is a result of neurodegeneration of retinal ganglion cells (RGCs) that form the inner-most layer of the retina and whose axons form the optic nerve which relays visual information to the brain (Calkins, 2012; Jonas et al., 2017).

In addition to glaucoma, autosomal recessive mutations in *ADAMTS10* cause Weill-Marchesani Syndrome (WMS), a rare connective tissue disorder characterized by short stature and ocular abnormalities including glaucoma (Faivre et al., 2003a; Dagoneau et al., 2004; Kutz et al., 2008). A clinically indistinguishable autosomal dominant form of WMS is caused by mutations in the fibrillin-1 gene (*FBN1*) (Faivre et al., 2003a; Faivre et al., 2003b; Sengle et al., 2012), suggesting overlapping function of ADAMTS10 and FBN1 proteins (Apte, 2009; Karoulias et al., 2020b). FBN1 assembles to form microfibrils, which are widely expressed components of the extracellular matrix that contribute to tissue biomechanics and are essential for the formation of elastic fibers (Ramirez and Sakai, 2010; Kielty, 2017). Microfibrils also act as extracellular storage sites for latent TGFβ, thereby controlling its activation and bioavailability (Charbonneau et al., 2004; Ramirez and Rifkin, 2009). Mutations in the genes encoding LTBP2 and ADAMTS17, both of which bind fibrillin-1, also cause WMS (Haji-Seyed-Javadi et al., 2012; Karoulias et al., 2020a). ADAMTS10 has been shown to bind FBN1 with high affinity and to colocalize with microfibrils and promote their formation (Kutz et al., 2011; Hubmacher and Apte, 2015; Cain et al., 2016; Matsuzaki et al., 2020). Recent studies with *Adamts10* mutant mice have shown that ADAMTS10 plays a role in a developmental switch of microfibrils from an immature form with predominant fibrillin-2 immunoreactivity, to a mature form with predominant fibrillin-1 immunoreactivity (Mularczyk et al., 2018; Wang et al., 2019; Wu et al., 2021). Mutations in FBN1 cause Marfan Syndrome, for which defective microfibril formation is a key component of pathogenesis, along with dysregulation of TGFβ signaling (Sakai et al., 2016). Therefore, disruption of microfibrils can lead to dysregulation of TGFβ signaling, which may have detrimental consequences that cause disease. TGFβ plays a role in neuronal development, including in the retina, where it participates in axonal growth and protects against apoptosis (Dunker et al., 2001; Braunger et al., 2013; Carrella et al., 2015; Tachibana et al., 2016).

ADAMTS10 is abundantly expressed in the developing mouse eye and up-regulation of *adamts10* mRNA has been shown in the developing zebrafish embryo (Brunet et al., 2015; Wang et al., 2019). In the present study, we found that targeting adamts10 expression in zebrafish embryos using a morpholino oligonucleotide (MO) resulted in abnormal RGC development accompanied by a large reduction in endogenous pSmad3-mediated TGFβ family signaling. Similar defective RGC development was induced by inhibition of TGFβ receptors. Our results suggest that Adamts10 plays a role in RGC development that is mediated by TGFβ family signaling.

## Materials and methods

### Zebrafish

Adult zebrafish (*Danio rerio*) were maintained in aquatic housing units on a monitored recirculating system at 26°C with standard light/dark (14/10 h) cycle. Experiments were performed using embryos from outcrosses of wildtype AB strain to a transgenic reporter line that expresses GFP under control of a *pou4f1* (*brn3a*) enhancer element, *Tg* (*pou4f1-hsp70l:GFP*)^*rw0110bTg*^ formerly *Tg* (*brn3a-hsp70l:GFP*)^*rw0110bTg*^ provided by Dr. Takanori Hamaoka and Dr. Hitoshi Okamoto of the Riken Brain Institute through the European Zebrafish Resource Center (Aizawa et al., 2005) or to a transgenic reporter line that expresses GFP in response to activation of a Smad3 binding element, *Tg*(*12xSBE:EGFP*)^*ia16Tg*^, generated by Dr. Francesco Argenton, Universita di Padova, obtained through the European Zebrafish Resource Center (Casari et al., 2014). Experiments using zebrafish embryos were conducted in accordance with the Association for Research in Vision and Ophthalmology statement for the Use of Animals in Ophthalmic and Vision Research and the Institutional Animal are and Use Committee of Vanderbilt University.

### RNA *in situ* hybridization

Zebrafish embryos or adult eyes were embedded in 20% sucrose/OCT and 7 μm-thick cryosections were made as described below for immunohistochemistry. RNA *In Situ* Hybridization was carried out on cryosections by the RNAscope technique (Wang et al., 2012) using a custom designed probe set for zebrafish *adamts10* mRNA and proprietary amplification reagents (mRNA *in situ* RNAscope 2.5 Chromogenic Assay, Advanced Cell Diagnostics, Hayward, CA) following the manufacturer’s protocol. The *adamts10* probe set was checked to avoid cross detection of the *adamts6* which has high homology with *adamts10*. A probe set for *DapB*, a bacterial gene, was used as negative control. Briefly, embryo sections were post-fixed in 4% PFA/PBS for 15 min at 4°C, dehydrated through gradient ethanol then treated with hydrogen peroxide for 10 min at room temperature. Target retrieval was conducted by submerging slides in retrieval solution for 5 min at 100°C. Following 30 min protease plus treatment at 40°C in a hybridization oven (HybEZ II Hybridization System, Advanced Cell Diagnostics), samples were hybridized with probes, subjected to signal amplification steps and then reacted with Fast Red. Bright field images were captured using a Nikon Eclipse microscope equipped with a ×40 objective.

### Embryo injections

Injections of 1–2 nl were made into the yoke adjacent to the embryo at the one-cell stage at approximately 12:00 p.m. To visualize injections, 0.025% phenol red was included in the injection solution. After injection, embryos were incubated at 29°C in egg water (Instant Ocean, Blacksburg, VA). At approximately 10:00 a.m. on post-injection days 1, 2 or 3 (referred to from here on as 24, 48, and 72 h post-fertilization (hpf), embryos were euthanized in 300 mg/L tricaine methanesulfonate, then fixed in 4% PFA/PBS. For fluorescence microscopy, 0.2 mM phenylthiourea (PTU) was added post-injection to prevent pigmentation.

### Morpholino oligonucleotide and *ADAMTS10* mRNA injections

MO obtained from Gene Tools (Philomath, OR), were reconstituted to 1 mM with RNase/DNase-free water and stored at room temperature as suggested by the manufacturer. Translation-blocking MO (5′-CCA​AAC​TCC​TCC​ACA​CCG​TTT​CCA​T-3′) targeting the translation initiation complex of *adamts10* mRNA (referred to from here on as *adamts10* MO) was injected at 4–5 ng/embryo. Human ADAMTS10 mRNA was transcribed from expression plasmids described previously (Kuchtey et al., 2011) using the *in vitro* T7 ultra mMessage mMachine™ kit with poly-A tail addition (Thermo Fisher Scientific). For MO rescue experiments, embryos were co-injected with 4 ng *adamts10* MO with 400 ng of either normal or G661R mutated human *ADAMTS10* mRNA.

### latent transforming growth factor *β* inhibition

To block TGFβ signaling, 100 µM SB431542 (Sigma), a concentration previously shown to drastically reduce reporter fluorescence of *Tg(12xSBE:EGFP)* embryos within 8 h after treatment (Casari et al., 2014), or vehicle control (DMSO) was added at 11 hpf to wells of 12 well-plates containing 10–12 GFP-positive *Tg* (*pou4f1-hsp70l:GFP*) or *Tg(12xSBE:EGFP)* embryos. This treatment protocol did not induce gross disruption of eye morphology, though treated embryos were slightly smaller than untreated (Supplementary Figure S1). At 48 hpf, embryos were euthanized in 300 mg/L tricaine methanesulfonate, fixed overnight in 4% PFA and stored in PBS at 4°C until examination by fluorescence microscopy.

### Imaging reporter fluorescence in whole embryos

GFP-positive embryos were dechorionated, fixed in 4% PFA/PBS for 2 h at room temperature or at 4°C overnight, then stored in PBS at 4°C. For imaging, embryos were placed ventral side up in 3% (w/v) methyl cellulose and fluorescence and brightfield images taken with a ×4 objective with a Nikon AZ100M fluorescence microscope.

### Immunohistochemistry

Embryos were embedded in 20% sucrose in optimal cutting temperature compound (OCT) as described by Barthel and Raymond. (1990) with modifications as follows: euthanized embryos were dechorionated then fixed in 4% PFA/5% sucrose in PBS for 1 h then incubated in increasing concentrations of sucrose (10%, 12.5%, and 15%) in PBS for 1 h and finally transferred to 20% sucrose/PBS and incubated overnight at 4°C. To facilitate visualization of embryos during orientation and sectioning, the 20% sucrose/PBS contained 0.002% toluidine blue. Embryos were then embedded and frozen in a 2:1 solution of 20% sucrose in OCT. Transverse cryosections 7 µm thick were cut with a CryoStar NX50 cryostat (Thermo Fisher Scientific), placed on glass slides and stored at -80°C until use.

For immunostaining, cryosections from the center of the eye were rehydrated with PBS before addition of blocking buffer (5% normal donkey serum/0.05% Triton X-100/PBS) for 1 h. Blocking buffer was removed and primary antibody solution in incubation buffer (3% normal donkey serum/0.05% triton X-100/PBS) was added. The antibodies used were 1:1,000 rabbit anti-GFP (Torrey Pines Biolabs) and 1:25 mouse anti-Isl1 (clone 40.2D6, Development Studies Hybridoma Bank, University of Iowa). After overnight incubation at 4°C in a humidified chamber, sections were washed 3 times in PBS for 10 min and incubated in 1:1,000 Alexa Fluor-donkey-anti-mouse-546 and 1:1,000 Alexa Fluor-donkey-anti-rabbit-488 (Invitrogen) in secondary incubation buffer (1% normal donkey serum/0.05% Triton X-100/PBS) for 1 h. Sections were then washed 3 times with PBS and mounted with Prolong Gold mounting fluid with DAPI (Invitrogen).

### Measurements and statistical analysis

Integrated fluorescence density of fluorescent embryo images, defined as the product of the area of the region of interest (ROI) and the mean gray value, was measured by drawing an ROI around the retina using the ImageJ polygon tool. 3D surface plots were generated from single-channel GFP images using the NIH ImageJ interactive 3D surface plot plug-in (http://rsb.info.nih.gov/ij/plugins/surface-plot-3d.html). Statistical analyses were performed in Graphpad Prism. Statistical tests and sample sizes are indicated in the figure legends.

## Results

### Ocular expression of adamts10 mRNA in zebrafish embryos

**FIGURE 1:**
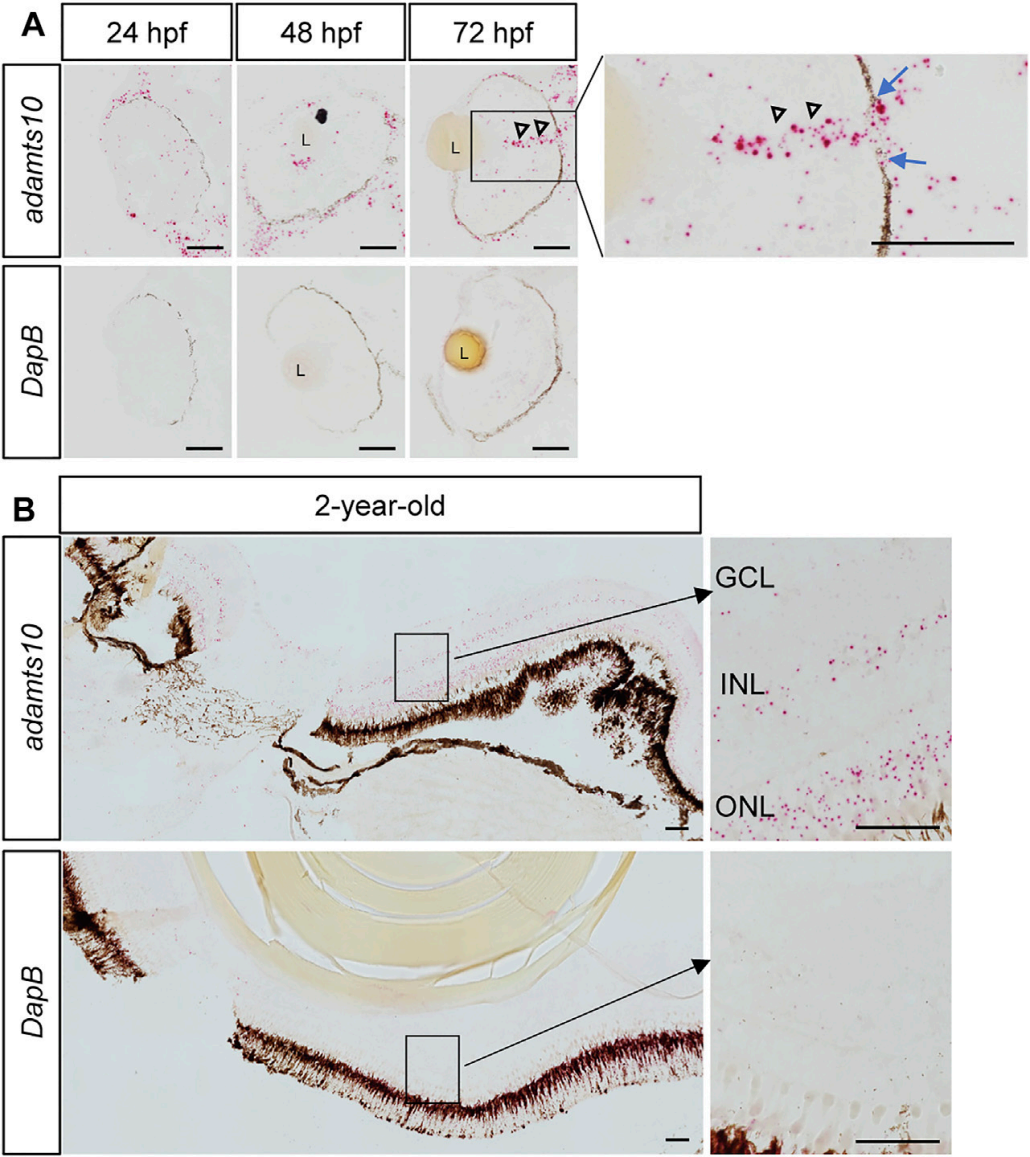
Ocular expression of *adamts10* mRNA in zebrafish. **(A)** Expression of *adamts10* mRNA (red dots, top row) was observed along the retinal pigment epithelium (dark brown/black structure) and dispersed throughout the retina of embryos at 24, 48, and 72 hpf. In 72 hpf eyes, robust signal (open arrowheads) extending from the inner retina and traversing a gap in the retinal pigmented epithelium (blue arrows, insert) is suggestive of optic nerve expression of *adamts10* mRNA. **(B)** In 2-year-old adult eyes, expression of *adamts10* mRNA was found in the outer nuclear layer (ONL), inner nuclear layer (INL), and ganglion cell layer (GCL). Hybridization probe against *DapB*, a bacterial gene, served as negative control on adjacent sections (bottom rows, **A** and **B**). Scale bar: 50 µm. L, Lens.

Expression of *adamts10* mRNA in the developing zebrafish embryos and in the adult zebrafish eye has been shown in a previous RT-PCR study (Brunet et al., 2015). By RNAscope *in situ* hybridization (Advanced Cell Diagnostics), we found *adamts10* mRNA expression in the eye of zebrafish embryos at 24, 48, and 72 hpf (Figure 1A) as well as in adult zebrafish 2 years of age (Figure 1B). Expression of *adamts10* mRNA was found along the retinal pigment epithelium and dispersed within the whole retina at 24, 48, and 72 hpf. In the eyes of 72 hpf embryos, robust signal extending from the inner retina and traversing the retinal pigment epithelium was suggestive of expression of adamts10 in the optic nerves. In 2-year-old adult eyes, *adamts10* mRNA was found in the outer nuclear layer, inner nuclear layer and ganglion cell layer. Given the retinal expression of *adamts10*, we chose to target its expression for reduction to investigate its role in RGC development.

### *Adamts10* morpholino oligonucleotide reduces *pou4f1* enhancer-driven GFP expression in the retina

**FIGURE 2:**
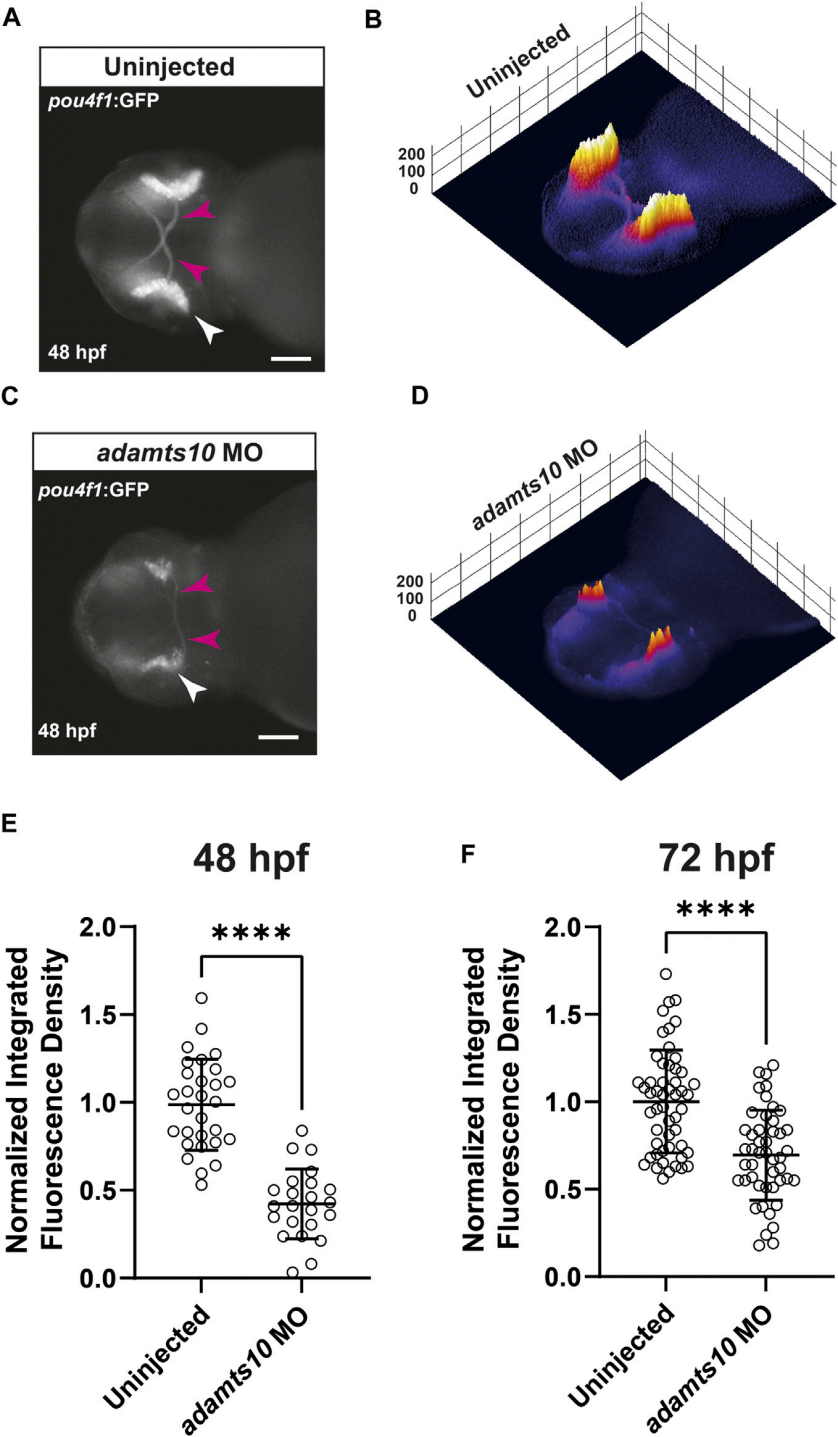
*Adamts10* MO reduces *pou4f1* enhancer-driven GFP fluorescence in zebrafish retinae. Representative fluorescent images of an uninjected **(A)** and an *adamts10* MO-injected **(C)** *Tg(pou4f1-hsp70l:GFP)* embryo at 48 hpf showing *pou4f1* enhancer-driven GFP fluorescence in the retina (white arrows) and optic nerves (magenta arrows, scale = 100 µm), with corresponding 3D surface plots of fluorescence intensity **(B,D)**. **(E)** Normalized integrated fluorescence density of uninjected and *adamts10* MO-injected embryos at 48 hpf (*n =* 29 and 23, respectively, ****p < 0.0001, Welch’s *t*-test). **(F)** Normalized integrated fluorescence density of uninjected and *adamts10* MO-injected embryos at 72 hpf (*n =* 52 and *n =* 47, respectively, ****p < 0.0001; Welch’s *t*-test).

Within the retina, Pou4f1 (also known as brn3a) is specifically expressed in ∼80% of developing and differentiated RGCs (Nguyen-Ba-Charvet and Rebsam, 2020). We used the *Tg(pou4f1-hsp70l:GFP)* line of zebrafish, which expresses GFP driven by a *pou4f1* enhancer element (Aizawa et al., 2005; Sato et al., 2015) to test the hypothesis that Adamts10 plays a role in RGC development. Embryos were injected at the one cell stage with *adamts10* MO or left uninjected. At 48 or 72 hpf, embryos were imaged to evaluate GFP fluorescence (Figure 2). In uninjected embryos at 48 hpf, GFP strongly labeled the retina and the optic nerve, which is composed of RGC axons (Figure 2A). As shown in 3D surface plots, GFP fluorescence in uninjected embryos appeared uniform in intensity along the retina, with faint fluorescence in the optic nerves (Figure 2B). However, in embryos injected with *adamts10* MO, GFP fluorescence was reduced and uneven, exhibiting patches of high and low intensity with barely visible optic nerves (Figures 2C,D). At 48 hpf, retinal fluorescence was reduced 50.4% in *adamts10* MO-injected embryos as compared to uninjected controls (*p* < 0.0001, Figure 2E). Decreased fluorescence was still evident at 72 hpf, although not as reduced as at 48 hpf, possibly due to dilution of MO with cell divisions, with a 26% reduction compared to uninjected control (*p* < 0.0001, Figure 2F). The reduction in GFP expression driven by a *pou4f1* enhancer suggests that injecting embryos with *adamts10* MO resulted in fewer RGCs and/or altered RGC development.

### Co-injection of *ADAMTS10* mRNA and *adamts10* morpholino oligonucleotide rescues *pou4f1* enhancer-driven GFP expression, but mutant G661R *ADAMTS10* mRNA does not

**FIGURE 3:**
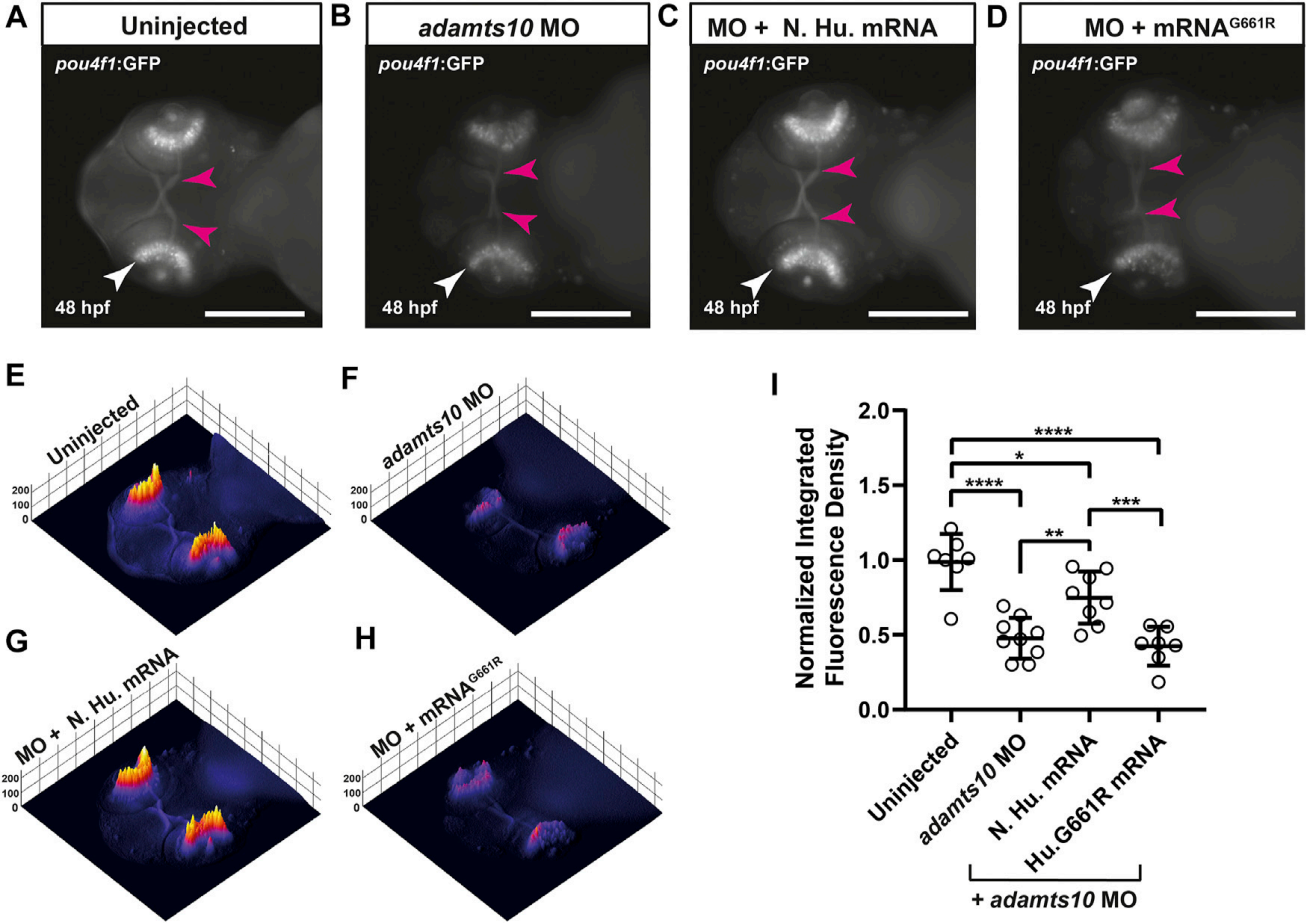
*Adamts10* MO-induced reduction in *pou4f1* enhancer-driven GFP expression is decreased with normal human *ADAMTS10* mRNA but not *ADAMTS10* ^*G661R*^ mutant mRNA*.* Representative fluorescent images of a *Tg(pou4f1-hsp70l:GFP)* embryo uninjected **(A)** injected with *adamts10* MO **(B)**, co-injected with *ADAMTS10* MO and normal human mRNA **(C)** and co-injected with *adamts10* MO and *ADAMTS10* ^*G661R*^ mRNA **(D)**, showing pou4f1 enhancer-driven fluorescence in the retina (white arrows) and optic nerve (magenta arrows); scale = 250 µm. Corresponding 3D surface plots showing fluorescence intensity for each condition **(E–H)** *.* Normalized integrated fluorescence density **(I)** of each condition (uninjected *n =* 7, *adamts10* MO-injected *n =* 9, normal human mRNA-injected *n =* 8 and *ADAMTS10* ^*G661R*^ mRNA *n =* 7, *p = 0.03, **p = 0.008,***p = 0.003, ****p < 0.0001; one-way ANOVA Tukey’s multiple comparisons test).

To confirm that MO-induced phenotypes were specific to *adamts10*, rescue experiments were performed in which *Tg(pou4f1-hsp70l:GFP)* embryos were co-injected with *adamts10* MO and human *ADAMTS10* mRNA (which is not targeted by the zebrafish *adamts10* MO). GFP fluorescence was compared at 48 hpf to embryos that were uninjected or injected with *adamts10* MO only. In uninjected embryos, the retina displayed strong fluorescence with staining of the optic nerve consistent with labeling of RGCs (Figure 3A). Fluorescence was uniformly distributed along the retina, as shown in the 3D surface plot (Figure 3E). Injection of *adamts10* MO alone resulted in a 51% decrease in GFP fluorescence in the retina compared with uninjected controls (*p* < 0.0001, Figure 3I), with a patchy distribution of low intensity GFP fluorescence in the retina (Figure 3F), similar to the MO experiment shown in Figure 2. Co-injection of *adamts10* MO with human *ADAMTS10* mRNA resulted in a milder 24% reduction of GFP intensity compared to uninjected embryos (*p* = 0.03), with a more uniform distribution of fluorescence intensity along the retina (Figures 3C,G,I), similar to uninjected embryos (Figures 3A,E). This partial rescue of the MO-induced retinal phenotype suggests that human *ADAMTS10* mRNA compliments a deficit of endogenous *adamts10* in MO-injected embryos. These results support the specificity of the MO effects for targeting of *adamts10* expression.

To determine if the glaucoma-causative G661R mutation affects the developmental function of ADAMTS10, rescue experiments were performed with G661R *ADAMTS10* mRNA. Co-injection of G661R mutant mRNA did not rescue the decreased GFP fluorescence which displayed retinal fluorescence intensity and pattern of expression similar to *adamts10* MO only injection, with a 56.3% reduction (*p* < 0.0001) in integrated fluorescence density (Figures 3D,H,I). The failure of G661R *ADAMTS10* mRNA to complement targeting of endogenous *adamts10* suggests that the glaucoma-causing mutation is deleterious for the developmental function of Adamts10.

### *Adamts10* morpholino oligonucleotide disrupts normal RGC development

**FIGURE 4:**
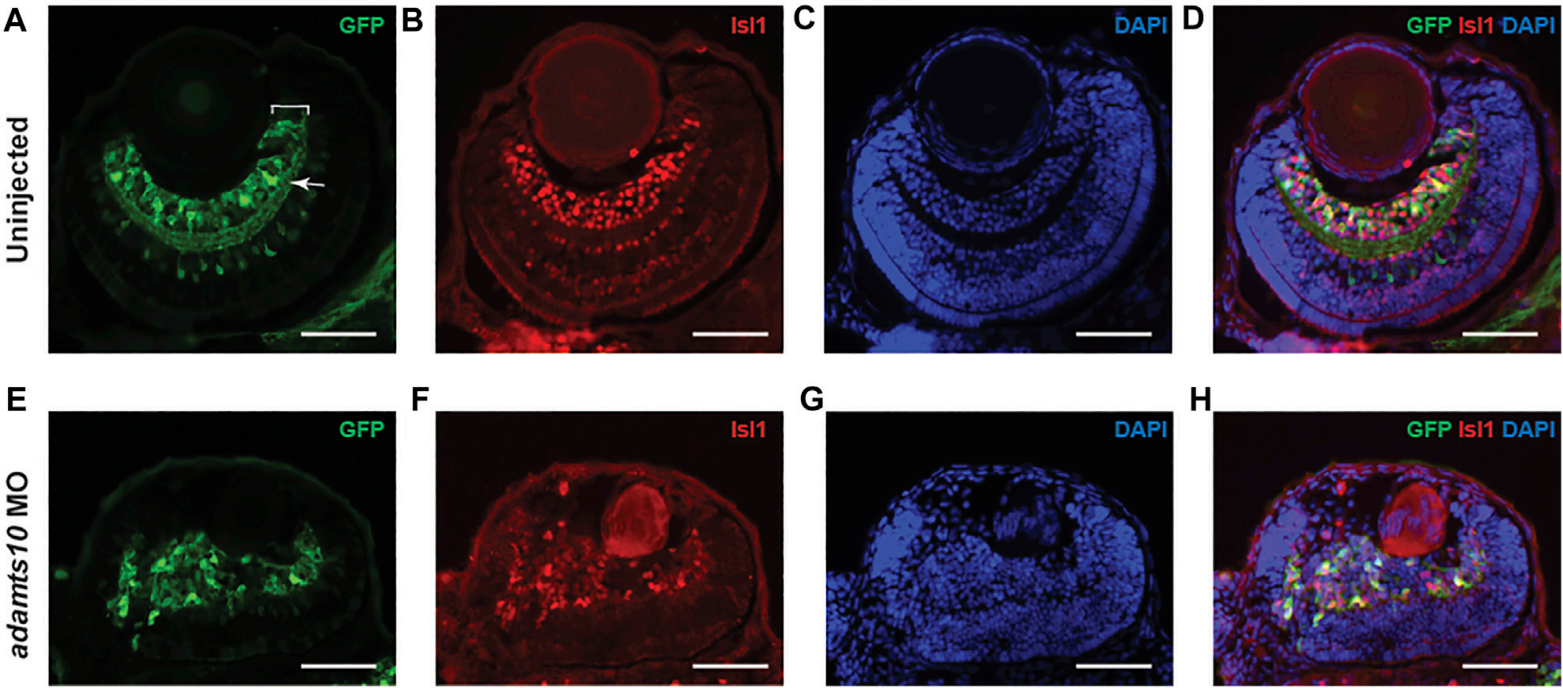
*A* *damts10* MO prevents proper formation of the ganglion cell layer. Representative images of stained sections from uninjected **(A–D)** and MO-injected **(E–H)** *Tg(pou4f1-hsp70l:GFP)* embryos at 72 hpf showing GFP staining **(A,E)**, Isl1 staining **(B,F)**, DAPI staining **(C,G)** and merged images **(D,H)**. In A, white bracket = ganglion cell layer, white arrow = inner plexiform layer. Scale = 40 µm).

To further investigate the effect of *adamts10* MO on retinal development, immunohistochemistry was performed on cryosections of 72 hpf *Tg(pou4f1-hsp70l:GFP)* embryos using an antibody against GFP and an antibody against Isl1, which is expressed in postmitotic RGCs and is required for RGC development (Pan et al., 2008). In uninjected controls, GFP-positive cell bodies with a range of fluorescence intensities defined the ganglion cell layer with radially oriented neurite processes (Figure 4A). The inner plexiform layer was clearly delineated by GFP-positive RGC dendritic processes. The ganglion cell and inner plexiform layers presented as well-defined layers of uniform thickness and semicircular shape adjacent to the lens. Isl1 immunostaining labeled cell bodies in the ganglion cell layer that overlapped with GFP positive cells (Figures 4B,D) confirming their identity as RGCs, though it appeared that more cells were labeled for Isl1 compared to GFP. These findings confirm that *pou4f1* enhancer-driven GFP specifically labels RGCs in the retina.

In *adamts10* MO-injected embryos, rather than forming a well-defined layer, GFP fluorescence labeled disordered groups of cells near the lens that often extended towards the outer retina with randomly oriented neurite processes and no defined inner plexiform layer (Figure 4E), demonstrating defective retinal lamination. Isl1 immunostaining overlapped with GFP-positive cells (Figures 4F,H), confirming their identity as differentiated RGCs, though the Isl1 fluorescence intensity appeared to be lower than that of eyes from uninjected embryos. Co-expression of Isl1 with Pou4f1 suggests that RGC development proceeds normally to some extent. However, the lamination defect suggests an inability of RGCs to migrate to their proper location.

### *Adamts10* morpholino oligonucleotide reduces pSmad3-mediated TGFβ family signaling in the retina

**FIGURE 5:**
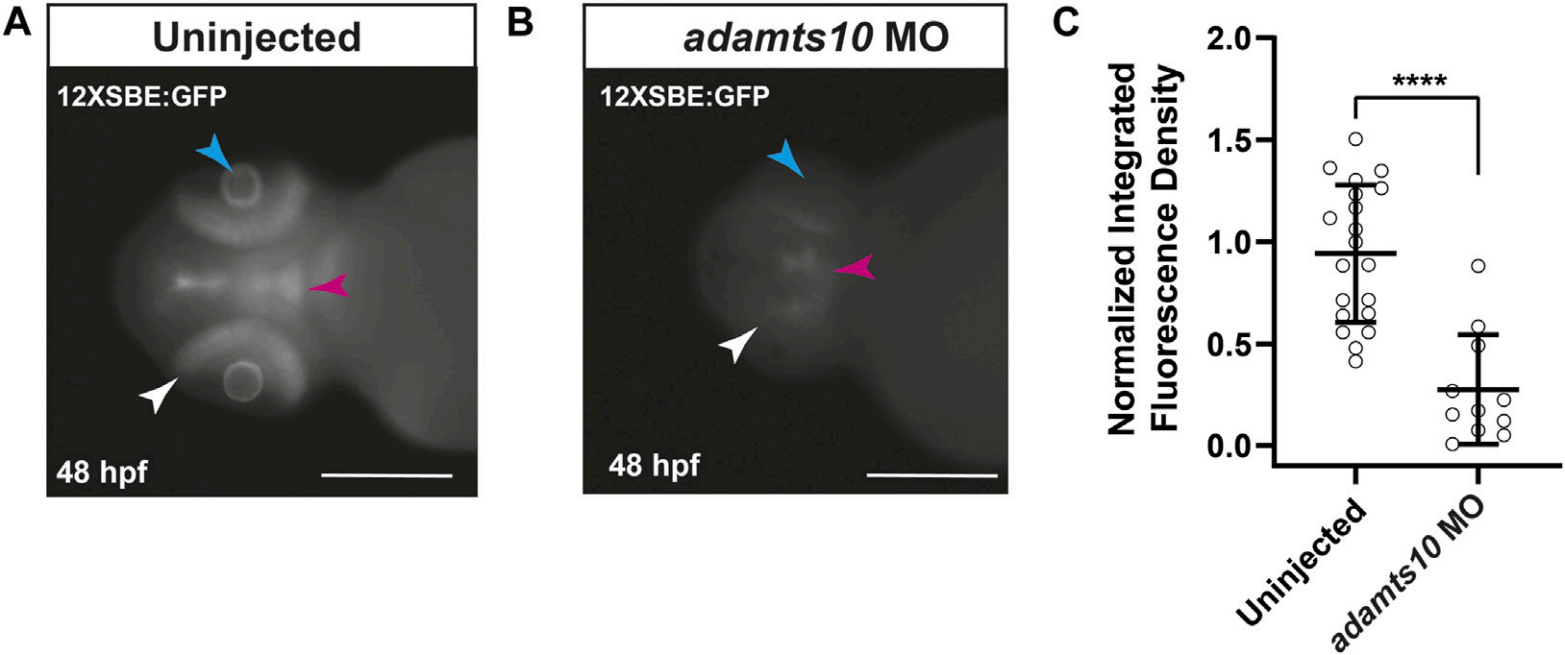
*Adamts10* MO reduces pSmad3-Mediated TGFβ family signaling in zebrafish retinae. Representative fluorescent images of an uninjected **(A)** and *adamts10* MO-injected **(B)** *Tg(12xSBE:EGFP)* embryo at 48 hpf showing fluorescence reporting of active pSmad3-mediated signaling in the retina (white arrows), brain (magenta arrows) and lens (cyan arrows, scale = 250 µm). Normalized integrated fluorescence density of uninjected, and *adamts10* MO-injected *Tg(12xSBE:EGFP)* embryos at 48 hpf [**(C)**, *n =* 21 and *n =* 11, respectively, ****p < 0.0001; Welch’s *t*-test].

ADAMTS10 plays a role in microfibril structure and function (Kutz et al., 2011; Sengle et al., 2012; Mularczyk et al., 2018; Wang et al., 2019). Since microfibrils regulate localization and activation of TGFβ signaling (Charbonneau et al., 2004; Ramirez and Rifkin, 2009), we tested the hypothesis that targeting Adamts10 expression would disrupt TGFβ family signaling. For these experiments, we used the *Tg(12xSBE:EGFP)* line of zebrafish which expresses GFP in response to activation of a pSmad3 binding element, thereby acting as a reporter of active TGFβ superfamily signaling (Casari et al., 2014). In uninjected embryos at 48 hpf, GFP fluorescence was found throughout the retina, as well as in the brain and lens of *Tg(12xSBE:EGFP)* embryos (Figure 5A), indicating constitutive TGFβ superfamily signaling in those tissues. Injection of *adamts10* MO resulted in a 66.6% reduction in fluorescence as compared to uninjected (Figures 5B,C, *p* < 0.0001). These results indicate that targeting Adamts10 expression drastically reduces constitutive pSmad3-mediated TGFβ superfamily signaling in the retina.

### TGFβ receptor inhibition recapitulates the effect of *adamts10* morpholino oligonucleotide on TGFβ family signaling in the retina

**FIGURE 6:**
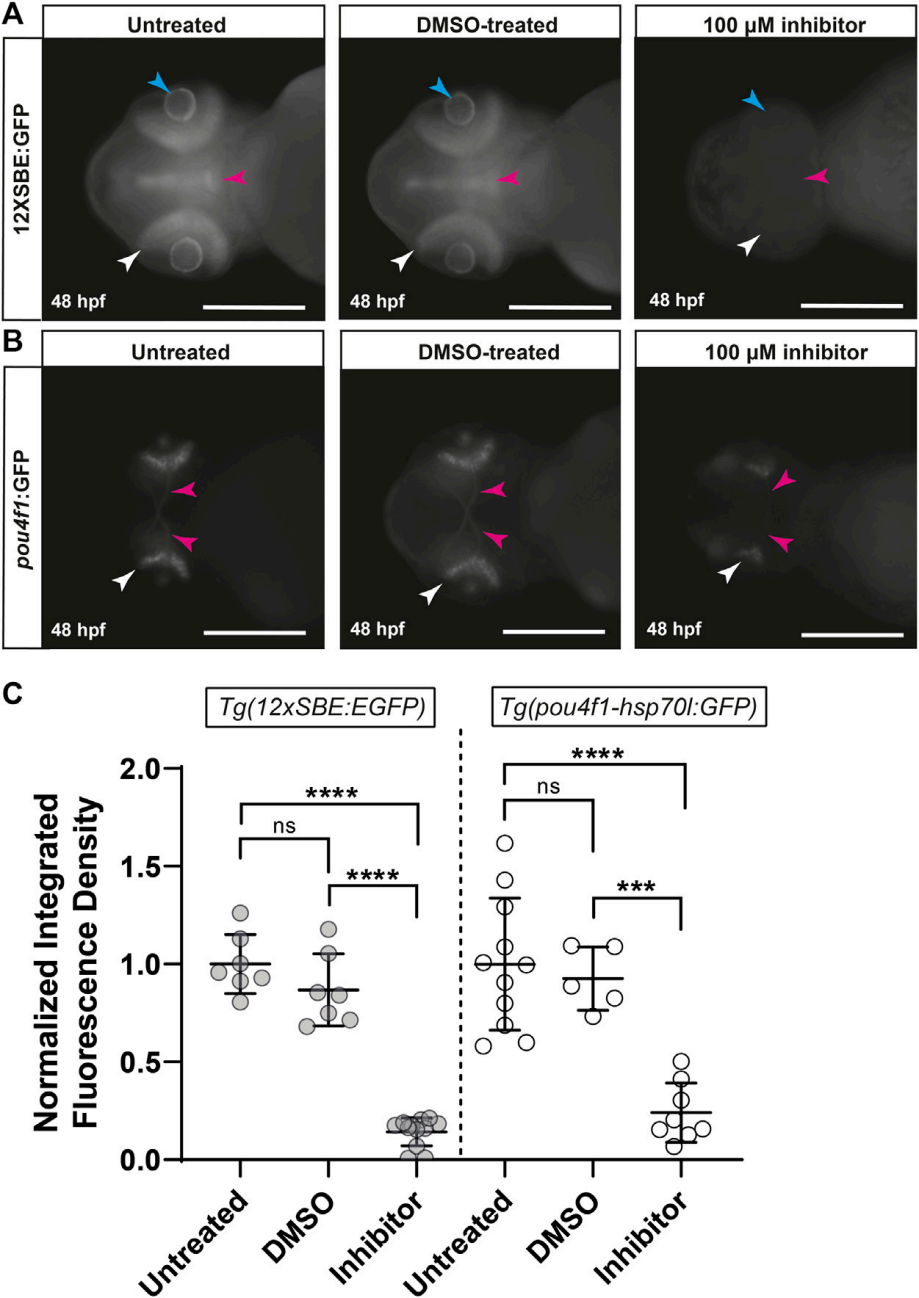
Inhibition of TGFβ signaling with SB431542 reduces *pou4f1* enhancer-driven GFP expression in the retina. Representative images of untreated, DMSO-treated and SB431542-treated *Tg(12xSBE:EGFP)* embryos at 48 hpf **(A)** showing GFP fluorescence reporting of pSmad3-mediated signaling in the retina (white arrows), brain (magenta arrows) and lens (blue arrows); scale = 250 µm. Representative images of untreated, DMSO-treated and SB431542-treated *Tg(pou4f1-hsp70l:GFP)* embryos at 48 hpf **(B)** showing *pou4f1* enhancer-driven GFP fluorescence in the retina (white arrows) and optic nerves (magenta arrows); scale = 250 µm. Normalized integrated fluorescence density **(C)** of each condition (*Tg(12xSBE:EGFP)*; untreated: *n =* 7, DMSO-treated: *n =* 7, SB431542-treated: *n =* 12: ****p < 0.0001, ***p = 0.0004, ns = non-significant; *Tg(pou4f1-hsp70l:GFP)*; untreated: *n =* 11, DMSO-treated: *n =* 5 and SB431542-treated: *n =* 8, ****p < 0.0001, ***p = 0.0004, ns = non-significant; one-way ANOVA Tukey’s multiple comparisons test.

The strong reduction in retinal pSmad3-mediated signaling by *adamts10* MO suggested that Adamts10 may exert its effect on RGC development through suppression of TGFβ signaling. To test this hypothesis, embryos were treated with an inhibitor of the TGFβ receptor, SB431542, at 11 hpf, a time at which the optic primordium has just formed (Kimmel et al., 1995), and fluorescence examined at 48 hpf. Embryos of the *Tg(12xSBE:EGFP)* line treated with SB431542 showed nearly complete reduction of pSmad3-driven GFP fluorescence, verifying effective inhibition of TGFβ signaling, while vehicle control (DMSO) had no effect (Figures 6A,C). To investigate the effect of TGFβ receptor inhibition on RGC development, *Tg(pou4f1-hsp70l:GFP)* embryos were treated with SB431542. Treatment with SB431542, but not vehicle control, strongly reduced *pou4f1* enhancer-driven GFP expression (76% reduction, *p* < 0.0001, Figures 6B,C), The reduction of RGC reporter fluorescence was similar to the reductions resulting from targeting adamts10 expression with MO (Figures 2, 3). These results support the hypothesis that effect of *adamts10* on RGC development is mediated by TGFβ.

## Discussion

A developmental role for ADAMTS10 is suggested by its association with WMS, a dysmorphic connective tissue disorder characterized by short stature and ocular phenotypes, including glaucoma (Faivre et al., 2003a). Recent studies suggest a role for ADAMTS10 in eye development by showing that *Adamts10* null mice retain their hyaloid vasculature into adulthood and are defective in a developmental fibrillin isotype switch (Mularczyk et al., 2018; Wang et al., 2019; Wu et al., 2021). Our study in zebrafish revealed for the first time that ADAMTS10 plays an important role in RGC development.

Mutations in *ADAMTS10* and in *ADAMTS17*, which is genetically and functionally highly similar to ADAMTS10 (Karoulias et al., 2020b) cause glaucoma in dogs (Kuchtey et al., 2011; Ahonen et al., 2014; Forman et al., 2015; Oliver et al., 2015). In humans, *ADAMTS8* is associated with key glaucoma endophenotypes (Wain et al., 2011; Springelkamp et al., 2014; Springelkamp et al., 2017), suggesting a role for ADAMTS genes in human glaucoma. Delineation of the biological functions of ADAMTS genes may lead to identification of novel targets for treatment of glaucoma patients. Homeobox genes such as *SIX6*/*SIX1*, *LMX1b* and *MEIS* (Carnes et al., 2014; Shiga et al., 2018), which play prominent roles in development, have been shown to be associated with glaucoma. *FOXC1*, a gene involved in ocular development, is associated with adult onset glaucoma in addition to congenital glaucoma (Souzeau et al., 2017). Also, *ATOH7*, a transcription factor expressed in post-mitotic RGC precursors and necessary for their development is strongly associated with optic disc cupping, an important risk factor for developing glaucomatous neurodegeneration (Ramdas et al., 2010). Our study suggests that *ADAMTS10* is another example of an ocular developmental gene that plays a role in glaucoma pathogenesis and suggests that the glaucoma-causative G661R mutation interferes with this developmental role.

Since glaucoma is defined as an optic neuropathy due to degeneration of RGC axons (Calkins, 2012), we focused on RGC development by looking at transcriptional activation of *pou4f1* (also known as *brn3a*) which is expressed early in post-mitotic RGC precursors and plays an important role in their development (Badea et al., 2009; Nguyen-Ba-Charvet and Rebsam, 2020). RGCs are the first cell type to differentiate in the developing retina, with RGC progenitors becoming postmitotic between 27 and 28 hpf in zebrafish (Hu and Easter, 1999; Avanesov and Malicki, 2010). Using the *Tg(pou4f1-hsp70l:GFP)* line of zebrafish that express GFP driven by a *pou4f1* enhancer element (Aizawa et al., 2005; Sato et al., 2015), we found that injecting embryos with a MO designed to block translation of *adamts10* mRNA resulted in reduced GFP expression in the retina at 48 and 72 hpf (Figures 2, 3), suggesting abnormal or delayed RGC development and/or a reduction in the number of RGC progenitor cells.

Since MOs can have non-specific effects, we performed rescue experiments and found that normal human *ADAMTS10* mRNA complimented the effect of co-injected *adamts10* MO (Figure 3). Interestingly, *ADAMTS10* mRNA carrying the glaucoma-causing G661R mutation was unable to compliment the MO effect, suggesting that this mutation interferes with the developmental function of *adamts10*, adding further support to the idea that defects in ocular development contribute to glaucoma pathogenesis. These results support specificity of the MO effect for targeting expression of *adamts10*, and support our conclusion that Adamts10 has a previously unknown role in retinal development.

In zebrafish, the RGC layer becomes discernable by 34–36 hpf and by 60 hpf, over 90% of the cells in the retina are postmitotic and the neuronal layers are established (Hu and Easter, 1999; Avanesov and Malicki, 2010). We performed immunohistochemistry on transverse sections from uninjected and *adamts10* MO-injected *Tg(pou4f1-hsp70l:GFP)* embryos using antibodies against GFP and Isl1 which is expressed in post-mitotic RGCs and plays key roles in RGC development (Wu et al., 2015). Expression of Isl1 in MO-injected embryos suggests that RGCs were able to undergo differentiation from retinal neural progenitors. However, Isl1 and GFP immunohistochemistry showed that RGCs from retinas of *adamts10* MO-injected embryos did not migrate to their proper position to form an ordered ganglion cell layer but instead formed disordered groups of cells (Figure 4). In addition, instead of forming an inner plexiform layer, RGC neuronal processes appeared randomly oriented. The disordered assembly of RGCs and lack of lamination suggests that targeting *adamts10* expression may deleteriously affect apical to basal migration of RGCs preventing formation of a normal ganglion cell layer (Icha et al., 2016), resulting in a retinal lamination defect.

Hyperactivated TGFβ signaling is well established in mouse models of microfibril deficiencies due to mutations in *FBN1* (Ramirez and Rifkin, 2009). Given that ADAMTS10 promotes microfibril formation (Kutz et al., 2011; Sengle et al., 2012), we hypothesized that reduction of Adamts10 expression would alter TGFβ signaling. TGFβ signal transduction is initiated by TGFβ binding to the receptors ALK5 (TβR1) and TβRII resulting in phosphorylation of Smad2 and Smad3 which form complexes with Smad4 that are translocated into the nucleus where they activate transcription (Massague, 2012). To test our hypothesis, we used the *Tg(12xSBE:EGFP)* line of zebrafish that expresses GFP in response to activation of a pSmad3 binding element. We found that uninjected *Tg(12xSBE:EGFP)* embryos expressed abundant GFP throughout the retina at 48 hpf (Figure 5), indicating constitutive pSmad3-mediated signaling. Injection of *adamts10* MO caused a large decrease in pSmad3-driven fluorescence, supporting our hypothesis that reduction of Adamts10 expression alters TGFβ signaling. It is interesting to note that the large decrease in pSmad3-mediated signaling resulting from disruption of Adamts10 expression is the opposite of the hyperactivation of TGFβ signaling found in fibrillin-1 deficiencies (Neptune et al., 2003; Ng et al., 2004; Habashi et al., 2006). Our result shows for the first time a role for Adamts10 in regulating pSmad3-mediated TGFβ family signaling and suggest a requirement for normal expression of *adamts10* to support constitutive TGFβ family signaling in the developing zebrafish retina.

A role for ADAMTS10 in TGFβ family signaling contrasts with the results of Mularczyk et al., who found that a premature termination mutation of *Adamts10* in mice did not affect pSMAD2/3-mediated TGFβ signaling but instead reduced pSMAD1/5/8-mediated bone morphogenic protein (BMP) signaling in mouse embryo fibroblasts (Mularczyk et al., 2018). Although we did not address BMP signaling since our focus was on TGFβ due to its importance in glaucoma, the effects of *ADAMTS10* deficiency could vary between tissues, developmental stages and species investigated. Another signaling role for ADAMTS10 described by Cain et al. that we did not investigate is its role in formation of focal adhesions and epithelial cell-cell junctions (Cain et al., 2016).

TGFβ is involved in neuronal development, including in the retina, in programmed cell death and in axon specification (Braunger et al., 2013; Carrella et al., 2015; Kaiser et al., 2020). This led us to form the hypothesis that the RGC developmental defect resulting from targeting Adamts10 expression is mediated by TGFβ. Consistent with our hypothesis, inhibiting the TGFβ receptor with SB431542 greatly reduced Pou4f1 enhancer-driven expression, similar to the effect of *adamts10* MO (Figure 6). However, it is important to note that in addition to the Alk5 TGFβ receptor, SB431542 inhibits the activin and nodal receptors, Alk4 and Alk7 (Inman et al., 2002). Although a TGFβ-mediated effect is consistent with a role for ADAMTS10 in microfibril formation, another possibility is that the pSmad3 signaling was initiated through activation of Alk4/Alk7 by Activin/Nodal. Smad3 signaling is activated only by the TGFβ family members TGFβ, activin or nodal (Pauklin and Vallier, 2015). Our results suggest that Adamts10 mediates its effects on retinal development *via* a pSmad3-mediated TGFβ family pathway.

Isl1 and Pou4f1 are expressed in early post-mitotic RGCs and are known to play important roles in their differentiation (Nguyen-Ba-Charvet and Rebsam, 2020). Expression of Isl1 and Pou4f1 reporter in retinas of adamts10 MO-injected embryos (Figure 4) suggest that production of post-mitotic RGCs was not prevented by targeting *adamts10* expression. This indicates that the primary defect of RGC development in *adamts10* MO treated embryos is likely a failure to migrate to the appropriate position resulting in a retinal lamination defect.

In summary, we have discovered a previously unknown role for Adamts10 in RGC development, possibly contributing to their apical to basal translocation and formation of an ordered ganglion cell layer. Our results suggest that the developmental role of Adamts10 is mediated by active TGFβ family signaling. In addition, our results show for the first time that Adamts10 is necessary for pSmad3-mediated constitutive TGFβ family signaling in the developing retina.

## Data Availability

The original contributions presented in the study are included in the article/Supplementary Material, further inquiries can be directed to the corresponding author.

## Abbreviations

hpf, hours post-fertilization; RGC, retinal ganglion cell.
