# Supplementary material for: Adamts10 controls transforming growth factor β family signaling that contributes to retinal ganglion cell development
Source: Front Mol Biosci. 2022 Sep 6;9:989851. doi: 10.3389/fmolb.2022.989851 (PMC9485804; doi:10.3389/fmolb.2022.989851)
Supplement: Supplementary file 1 [file Presentation1.PPTX]

## Slide 1
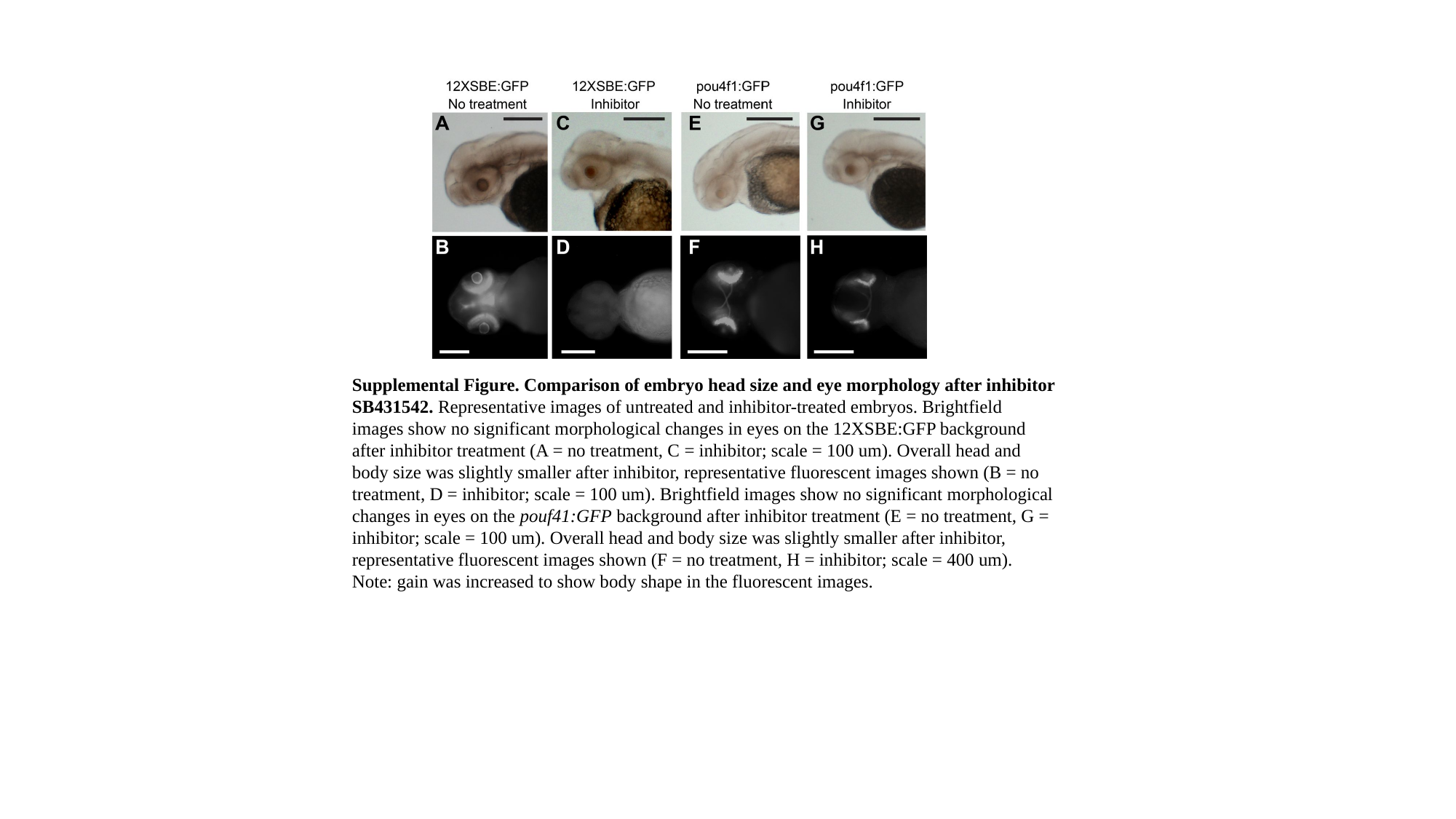

Supplemental Figure. Comparison of embryo head size and eye morphology after inhibitor SB431542. Representative images of untreated and inhibitor-treated embryos. Brightfield images show no significant morphological changes in eyes on the 12XSBE:GFP background after inhibitor treatment (A = no treatment, C = inhibitor; scale = 100 um). Overall head and body size was slightly smaller after inhibitor, representative fluorescent images shown (B = no treatment, D = inhibitor; scale = 100 um). Brightfield images show no significant morphological changes in eyes on the pouf41:GFP background after inhibitor treatment (E = no treatment, G = inhibitor; scale = 100 um). Overall head and body size was slightly smaller after inhibitor, representative fluorescent images shown (F = no treatment, H = inhibitor; scale = 400 um). Note: gain was increased to show body shape in the fluorescent images.
